# Editorial: Mass Spectrometry for Adductomic Analysis

**DOI:** 10.3389/fchem.2019.00794

**Published:** 2019-11-21

**Authors:** Marcus S. Cooke, Chiung-Wen Hu, Mu-Rong Chao

**Affiliations:** ^1^Oxidative Stress Group, Department of Environmental Health Sciences, Florida International University, Miami, FL, United States; ^2^Biomolecular Sciences Institute, Florida International University, Miami, FL, United States; ^3^Department of Public Health, Chung Shan Medical University, Taichung, Taiwan; ^4^Department of Occupational Safety and Health, Chung Shan Medical University, Taichung, Taiwan; ^5^Department of Occupational Medicine, Chung Shan Medical University Hospital, Taichung, Taiwan

**Keywords:** DNA, protein, adductomics, adducts, genomic instability, mass spectometry, exposure, disease

Individuals are continually exposed to physicochemical agents from the internal and external environments. The majority of these exposures act, at least in part, via the modification of DNA, or proteins, generating a wide range of adducts. DNA (Cooke et al., 2003), and some forms of protein (Barrera et al., 2015), damage impact cellular function, and play a critical role in the pathogenesis of, arguably, all major human diseases. Therefore, the assessment of DNA and protein damage is central to a wide variety of biomedical, and related fields. However, the majority of the literature describes the use of targeted analyses of damage, measuring only single, or a few, adducts, and while of value, this approach fails to reflect the totality of adducts, and therefore exposures.

Adductomics refers to the totality of cellular adducts associated with, to date, proteins (Rappaport et al., 2012), or DNA (Kanaly et al., 2006), and both are considered in the present collection. These untargeted approaches provide a most comprehensive profile of the damage derived from combinations of known and/or unknown exposures and, to some extent, the biological response(s) to such exposures (Chang et al., 2018).

In their mini review, Chen and Li focus on catechol quinone (CQ)-derived protein adducts. The presence of the CQ motif in many biologically relevant molecules (including products of endogenous metabolism, certain foods, drugs, and environmental pollutants), and the fact that CQ-adducted proteins may exhibit cytotoxicity or biological functions different from their unmodified forms, make this class of adducts highly worthy of study, but this is technically challenging. Chen and Li advocate the protein adductomics approach for studying CQ-adducted proteins; for both their characterization, and quantification in blood, as biomarkers of exposure. The authors also provide valuable insight into some of the technical challenges that these analyses present.

In an exciting potential medical application of protein adductomics, Geib et al. characterize the specific adducts formed when the products of acetaminophen (APAP; paracetamol) interact with key cellular defenses against toxicity (the glutathione *S*-transferases). APAP-induced hepatotoxicity is the most common cause of acute liver failure in the Western world, making it a major public health problem. Metabolism of APAP generates a reactive intermediate which can react with glutathione and protein thiols. LC-MS/MS analysis revealed seven modified cysteine sites, including two unique sites, demonstrating the power of both untargeted and targeted approaches.

The identification of both DNA and protein adducts, discovered during adductomics analysis, is challenging due to the large size of the datasets generated, and the lack of data processing approaches. Whereas, proteomics analysis has benefited from technological advances in mass spectrometry, and bioinformatics, this has yet to fully translate to protein (and DNA) adductomics. Nunes et al., inspired by the workflows used in metabolomics, developed a novel analysis approach for the identification of covalently-modified peptides. The strategy was tested by the analysis of histone-derive adducts from HepG2 and THLE2 cells treated with glycidamide, and the results compared against standard methodology. Crucially, the new strategy identified adducts not seen with the conventional approach, and appears likely to advance the protein adductomics field further.

Although cellular DNA adductomics is less well-established than protein, the field is gaining momentum. In this collection, two reports illustrate the strengths of DNA adductomics, the potential for it to significantly advance our understanding of the links between environmental exposures and disease, and the scope for further methodological and application advances.

Carrà et al. aimed to develop a screening methodology for all known endogenously generated DNA adducts using high resolution data-dependent scanning, an extensive MS^2^ fragmentation inclusion list of all known endogenous adducts, and neutral loss MS^3^ triggering to profile all DNA modifications. Crucially, the high sensitivity required to detect some of these low abundance adducts was achieved by decreasing extraneous background signal through pre- and post-DNA hydrolysis steps, and the optimization of several instrument parameters. The success of these improvements was demonstrated in an animal model of lung carcinogenesis. This is a prime example of a concerted approach to refine existing methodology, which can benefit the entire field. Adoption of the lessons learnt from this work should further advance the quality of data, and hence biological significance, achieved by cellular DNA adductomics.

*In vitro* genotoxicity is a first line test when screening new chemical agents, and is generally performed using high throughput assays. However, confirmation of DNA adduct identities can be a time-consuming and expensive process. For the first time, Takeshita and Kanaly describe an *in vitro* RNA adductomics approach and, in combination with *in vitro* DNA adductomics, proposed that these combined may address this issue. This innovative approach was evaluated using Hep G2 cell lines exposed to benzo[*a*]pyrene, and the modified 2′-deoxynucleosides or ribonucleosides studied by LC/ESI-MS/MS, through neutral loss targeting of the [M + H]^+^ > [M + H – 116]^+^ or [M + H]^+^ > [M + H −132]^+^ transitions, respectively. The significance of RNA adducts is not fully understood, and while translational mutagenesis has been reported (Stirpe et al., 2017), it is unclear whether or not RNA is repaired (Yan and Zaher, 2019). Consequently, the analysis of RNA adducts is not widespread (although the analysis of urinary 8-oxoguanosine, for example, is becoming more common; Shih et al., 2019). RNA adductomics extends the field to include this class of cellular biomolecule, and we predict that it is likely to shed further light on the biology of RNA damage. Also, consideration should be given to classes of adducts not routinely detectable by current adductomics methodology, such as DNA-DNA crosslinks (Hu et al., 2019), and DNA-protein crosslinks (“crosslinkomics”), the latter being an interesting intersection of the DNA and protein adduct field.

There will always be a place for targeted analyses of adducts. However, with a growing number of means to minimally- (Hwa Yun et al., 2018), or non-invasively (Cooke et al., 2018) undertake adductomics analysis of human samples, the increasing availability of methodology for the more routine LC-QqQ-MS/MS platform, as costs decrease, and data analysis becomes more routine/simpler, we envisage adductomics becoming a more routine procedure.

This collection covers topics representative of a number of key research areas in the field of adductomics. The highly innovative work of these authors, and others, will continue to accelerate adductomic methodologies, and discoveries, all to the benefit of human health.

## Author Contributions

All authors listed have made a substantial, direct and intellectual contribution to the work, and approved it for publication.

### Conflict of Interest

The authors declare that the research was conducted in the absence of any commercial or financial relationships that could be construed as a potential conflict of interest.
